# Mandibular second molar impaction: introducing a novel and validated 3D classification system

**DOI:** 10.1186/s12903-024-05006-x

**Published:** 2024-10-10

**Authors:** Selene Barone, Lucia Cevidanes, Tecla Bocchino, Ambra Michelotti, Massimo Borelli, Amerigo Giudice

**Affiliations:** 1 Department of Health Sciences, Magna Graecia University of Catanzaro, Catanzaro, Viale Europa – 88100 Italy; 2 Department of Orthodontics and Pediatric Dentistry, University of Michigan, Ann Arbor, USA; 3 Department of Neurosciences, Reproductive Sciences and Oral Sciences, Section of Orthodontics and Temporomandibular Disorders, University of Naples Federico II, Naples, Via Sergio Pansini 5, 80100 Naples, Italy

**Keywords:** Mandibular second molar impaction, Three-dimensional assessment, Degree of difficulty, 3D classification system, Surgical-orthodontic assessment, Mandibular molar eruption disturbance

## Abstract

**Background:**

Mandibular second molar (M2M) impaction is a clinically significant manifestation of eruption disturbance in dental development. The primary aim of this study was to investigate the impact of the three-dimensional (3D) characterization on clinical and therapeutic decisions for M2M impaction. The secondary aim was to introduce a validated 3D classification system incorporating both surgical and orthodontic parameters.

**Methods:**

Bidimensional (2D) and 3D radiological records of 15 impacted M2M were collected and deidentified. Ten experienced clinicians (5 oral surgeons;5 orthodontists) categorized each case, first based on 2D records and then with 3D scans. The degree of orthodontic and surgical difficulty in treating impacted M2M was evaluated using a novel classification system based on anatomical and radiological features. The primary outcome variable was the assessment of differences in diagnosis and decision-making protocol using 2D or 3D records, where clinical relevance ranged from 0 to 4. The secondary outcome variable was the validation analysis of the proposed 3D classification system to determine the concordance among the clinicians. Descriptive statistics and multivariable inferential analysis based on Akaike information criterion (AIC) were performed (α = 0.05).

**Results:**

3D examination allowed a better visualization of M2M impaction with higher clinical relevance for diagnosis of M2M root relationship to alveolar nerve and lingual plate, height to alveolar crest, depth, and inclination relative to the first molar and position relative to the third molar (range:2.69–3.43). The proposed 3D classification of M2M impaction changed clinical decisions regarding surgical-orthodontic approach, biomechanics, patient education, and treatment time estimate (range:2.59–3.33). In the validation analysis of the classification, no evidence of inter- or intra-group (surgeon/orthodontist) bias in score attribution occurred: the model with the minimum AIC was the null model (AIC = 718.04).

**Conclusion:**

3D evaluation of impacted M2Ms could enhance diagnostic accuracy, and a classification system was proposed and validated by a group of experienced surgeons and orthodontists with high concordance.

**Supplementary Information:**

The online version contains supplementary material available at 10.1186/s12903-024-05006-x.

## Introduction

Mandibular second molar (M2M) impaction is a clinically significant manifestation of eruption disturbance in dental development, as second molars are of great importance for the normal development of the dentition and coordination of the posterior vertical dimension [1–5]. While M2M impaction occurs in 0.03% to 0.65% of adolescents, with a peak at 4.6% among those requiring orthodontic treatment, recent studies suggest an increasing trend in M2M impaction that emphasizes the necessity for a thorough understanding and effective management of this condition [1, 6–8]. The onset of impacted M2M is typically identified between 11 to 14 years of age, with a predilection for males. The altered eruption could be influenced by puberty-related bone growth and hormonal modifications that are common at this age [7]. Diagnosing M2M impaction can be challenging due to the absence of symptoms and delayed primary teeth exfoliation [9]. For the etiology, various theories have been proposed, involving internal factors like ectopic follicle position and external factors such as deficits in retromolar space, primary dentition disorders (ankylosis or premature loss), neighboring permanent teeth alterations, and obstacles in the eruption path [6, 7, 9–14]. Disturbances can also arise from supernumerary teeth, ectopic positions of the third molar, and local pathological bone lesions like dentigerous cysts or tumors, hindering the guidance for M2M eruption [6, 9, 10, 12]. Additionally, a genetic predisposition may be associated with M2M impaction, considering specific craniofacial characteristics such as a retrognathic mandible and a smaller mandibular gonial angle [8, 14, 15].

Eruption alterations can significantly impact the relationship of the mandibular second molar with surrounding structures, and the utilization of Cone Beam Computed Tomography (CBCT) offers a three-dimensional evaluation and provides enhanced visualization, aiding in precise diagnostics across spatial axes [1]. In the coronal view, a vertically-oriented impaction may bring the M2M into close proximity with the alveolar nerve, influenced by infraocclusion and root development [3–5, 10]. Axial views reveal lingual impactions, potentially impacting the proximity to the lingual nerve near the cortical plate [10]. A sagittal perspective uncovers various implications, including distal inclination affecting the mandibular ramus and third molar follicle, and mesial angulation or severe horizontal impaction resulting in root resorption and periodontal damage to the first permanent molar [10]. To date, case reports or case series are the most frequent articles in literature about eruption disturbance of M2M [16–18]. Although excellent results have been achieved in treating impacted M2Ms, there is still no standardized classification system to accurately characterize their difficulty. While some evaluations, such as the Pell and Gregory classification, are commonly used in clinical practice, they are not specifically designed for impacted M2Ms and do not account for the challenges of 3D assessment. This lack of a dedicated system limits the ability to consistently assess and plan for varying degrees of impaction complexity.

The primary aim of this study was to investigate the impact of the 3D characterization on clinical decisions and therapeutic approaches for M2M impaction. The secondary aim was to introduce a validated three-dimensional classification system. This system incorporates both surgical and orthodontic parameters to comprehensively assess the degree of difficulty associated with this eruption disorder.

## Materials and methods

### Study design and ethics declaration

The study was conducted as an observational single-center cohort study in accordance with the Declaration of Helsinki. The Ethics Committee of the Calabria region – central area section approved the study (n.143/2018). To collect radiological data for scientific analysis, the informed consent was obtained from all the participants in the study.

### Study sample and data availability declaration

This investigation involved secondary data analysis of available radiological records acquired for clinical purposes of patients with impacted M2Ms. The inclusion criteria were patients with either unilateral or bilateral M2M impaction, having available radiographic records: orthopantomography for bidimensional (2D) evaluation and CBCT scans for 3D assessment. The exclusion criteria were patients aged < 12 years (below the physiological eruption age of the M2M), with craniofacial deformities or syndromes, periapical lesions in the mandibular first molars, history of traction of the impacted M2M, and poor quality CBCT scans.

From the internal databases, radiographic records were selected and deidentified for the validation analysis. The sample included 10 patients (7 males and 3 females; mean age: 19.4 ± 4.4) with 15 impacted M2Ms: 5 patients with bilateral and 5 patients with unilateral M2M impactions.

### Data collection method

A classification of degree of difficulty was implemented based on the anatomical and radiological features of the impacted mandibular second molar. The difficulty degree was assessed considering surgical and orthodontic items, because of their intersection in the treatment of impacted M2M. The position of each of the included 15 impacted M2Ms was categorized according to the proposed classification system. Ten clinicians with more than 20 years of experience were enrolled for this validation analysis, distinguishing two groups about their specialty: five orthodontists and five oral and maxillofacial surgeons. First, clinicians were given access to the deidentified 2D records, and then asked to independently complete a questionnaire regarding the diagnosis and treatment planning for each case of impaction (Supplementary Fig. 1). After completing their questionnaire, the 3D records of the same case were shown to the clinicians and a second survey aimed to analyze if the 3D characterization could have a role in modifying the diagnosis or treatment approach (Supplementary Fig. 2). The diagnostic relevance was assessed for each item of the questionnaire with five different levels of significance: 0 (No importance), 1 (Low importance), 2 (Mild importance), 3 (Moderate importance), 4 (High importance).

### Degree of difficulty of M2M impaction using anatomical and radiologic features

Based on the anatomical and radiological features that were reviewed, a 3D classification system for M2M impaction was proposed. Considering the close cooperation between oral surgeons and orthodontists in treating this condition, the classification focused on both the technical difficulties for surgeons, and the biomechanics challenges related to M2M orthodontic traction. The classification evaluates the mandibular second molar in the three spatial axes, highlighting its relation to specific anatomical structures: mandibular canal, alveolar crest, first permanent molar, third permanent molar, buccal and lingual plates. Each parameter has a score ranging between 1 and 3, according to simple, moderate, or complicated condition. To evaluate M2M impaction, two measurements scales were introduced: an ordinal numerical scoring and an ordinal categorical classification. The ordinal categorical classification has been introduced exploiting a resampling approach, with a view to overcome judgement variability among operators. After considering all the seven parameters, the null distribution of possible scores sum was bootstrapped by *N* = 50.000 times. After computing the null distribution tertiles, the difficulty degree has been categorized as simple, moderate, or complicated.

**Fig. 1:**
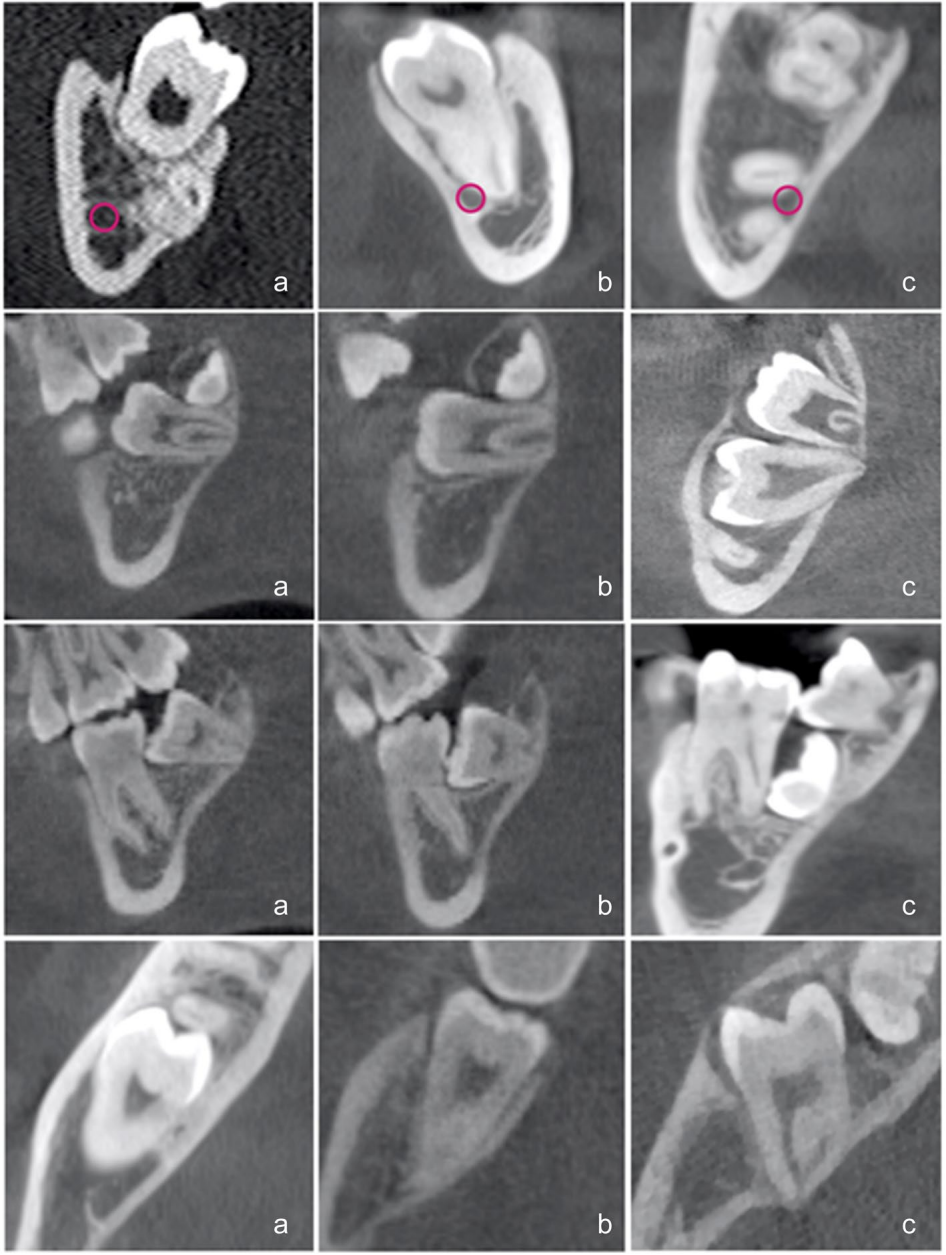
Three-dimensional radiological assessment of impacted M2Ms according to the surgical items of the proposed classification of difficulty: simple (in the first column; a), moderate (in the second column; b), and complicated (in the third column; c). Apico-coronal axis: the first two lines show M2M position related to mandibular canal and alveolar crest. Mesio-distal axis: the third line evaluates M2M position related to the first molar. Bucco-lingual axis: the last line shows M2M position related to the cortical plates

**Fig. 2:**
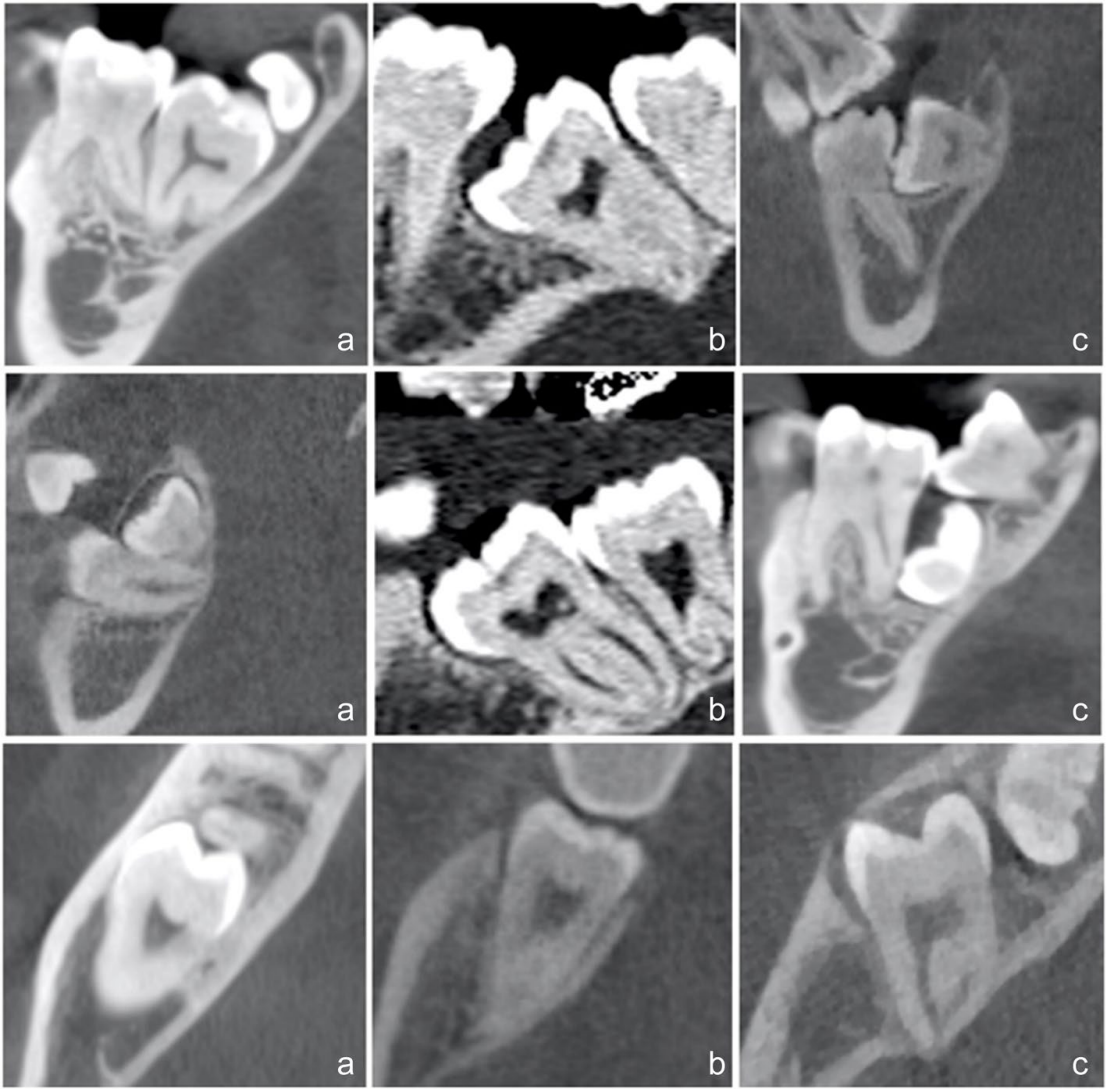
Three-dimensional radiological assessment of impacted M2Ms according to the orthodontic items of the proposed classification of difficulty: simple (in the first column; a), moderate (in the second column; b), and complicated (in the third column; c). Apico-coronal axis: the first line shows M2M inclination related to the long axis of the lower first molar. Mesio-distal axis: the second line evaluates M2M position related to the third molar. Bucco-lingual axis: the last line shows M2M position related to the cortical plates

The degree of difficulty for managing the impacted mandibular second molar was summarized in Table 1 (Fig. 1–2).

**Table 1:** Proposal of classification for degree difficulty of impacted mandibular second molars

Surgical Difficulty			
	Simple	Moderate	Complicated
Apico-coronal axis			
Relationship with the mandibular canal (MCs)	Distance between the roots and the mandibular canal	Relationship between the roots and the mandibular canal	Mandibular canal is between the roots
Relationship with the alveolar crest (ACs)	Partially impacted (complete eruption of the distal and mesial cusps of the crown)	Partially impacted (partial eruption of the distal cusps of the crown)	Bone impaction
Mesio-distal axis			
Relationship with the first molar (M1Ms)	M2M crown is above the amelocementitious junction of M1M	M2M crown is between the amelocementitious junction of M1M and the apical third of M1M roots	M2M crown is at the apical third of M1M roots
Buccal-lingual axis			
Relationship with the cortical plates (CPs)	M2M has a relationship with the buccal cortical plate	M2M has a relationship with the buccal and lingual cortical plates	M2M is transversally positioned contacting buccal and lingual cortical plates
Orthodontic Difficulty			
	Simple	Moderate	Complicated
Apico-coronal axis			
Angle between the long axis of M1M and M2M (M2M1o)	M2M is mesially inclined with acute angle (< 45°)	M2M is mesially inclined with obtuse angle (> 45°)	M2M is in horizontal position (angle = 90°)
Mesio-distal axis			
Relationship with the third molar (M3Mo)	M3M is completely distal to M2M	M3M is disto-coronally located respect to M2M	M3M is mesially drifted and located in the region of M2M
Buccal-lingual axis			
Relationship with the cortical plates (CPo)	M2M is parallel to cortical plates	M2M is oblique to the cortical plates	M2M is perpendicular to the cortical plates

*M2M* mandibular second molar, *M1M* mandibular first molar, *M3M* mandibular third molar, *MCs* mandibular canal-surgical item, *ACs* alveolar crest-surgical item, *M1Ms* mandibular first molar-surgical item, *CPs* cortical plate-surgical item, *M2M1o* angulation between M2M and M1M-orthodontic item, *M3Mo* mandibular third molar-orthodontic item, *CPo* cortical plate-orthodontic item

In the apico-coronal axis, the M2M is initially analyzed in relation to its depth of impaction. The therapeutic approach is considered more and more difficult with increasing the distance from the alveolar crest. The second factor involves the relationship between M2M and the mandibular canal. The contact between the root apices with the mandibular canal can increase the risk of damage. Orthodontically, apico-coronal axis allows the assessment of M2M inclination in relation to the first molar, calculating the angle between the long axes of these two teeth. A parallelism or mild inclination are recognized as the easiest conditions, on the contrary, the difficulty increases with a more horizontal position of M2M.

In the mesio-distal axis, M2M impaction is assessed in relation to the permanent first molar: M2M crown can be above or below the cementoenamel junction in simple or moderate conditions, while M2M crown is at the same level of the apical roots of the first molar in the complicated cases. From the orthodontic point of view, mesio-distal direction correlates M2M impaction with the position of the mandibular third molar (M3M): M3M could be completely distal to the impacted tooth as the easiest condition or, alternatively, moderate to severe conditions show a reduced retromolar space that is partially or totally occupied by the third molar.

In the bucco-lingual axis, both surgical and orthodontic treatment challenges concern the relationship between M2M and the cortical plates, but from a different perspective. Surgically, M2M has an increasing difficulty if it is 1) buccally positioned; 2) in the middle between buccal and lingual plates; 3) transversally positioned. Orthodontically, M2M impaction depends on if it is parallel to the cortical walls and increasing the difficulty if it is oblique or completely perpendicular to them.

### Study variables

The primary predictor variable was the specialty of the clinicians: orthodontists or oral and maxillofacial surgeons.

The primary outcome variable was the assessment of any difference in diagnosis and decision-making protocol using 2D or 3D radiological records for M2M impaction, where clinical relevance was ranked from 0 to 4.

The secondary outcome variable was the validation analysis of the proposed 3D classification system to determine the concordance among the clinicians.

### Statistical analysis

Statistical analysis was blindly conducted using the software R language. Descriptive statistics recorded absolute and relative frequencies for categorical data, mean and standard deviation for continuous quantitative variables. Ordinal variables were described by median, quartiles, and range. The concordance among clinicians regarding the items of the questionnaire was reported. The concordance among clinicians about the items of the questionnaire were reported. For the validation analysis, a multivariable inferential analysis was conducted using generalized linear models to analyze various characteristics of the same impacted M2M (depth of impaction, relationship with the mandibular canal, inclination relative to the first molar, position relative to M1M and M3M, and relationship with the cortical plates). The secondary outcome (the validation of the proposed classification) was considered as a Poisson random variable to account for the number of discrete events (concordance among clinicians on each specific item). Model selection was performed using the Akaike Information Criterion (AIC). Starting from an additive maximal model, non-significant terms were observed. A top-down simplification procedure was then employed to achieve a model where all predictors are significant, ensuring the AIC criterion is as low as possible. This approach led to the identification of the minimal adequate model. In all inferential analyses, α = 0.05 has been assumed as significance level.

## Results

The 2D analysis of M2M impaction showed that the third molar was considered an obstacle for M2M eruption, with 83% of clinicians in agreement. Most clinicians (88%) agreed on the necessity of a CBCT for a deeper examination. Concerning the possibility of tooth recovery, there was high concordance among the clinicians (82%), with the treatment time for M2M repositioning being more than 6 months (66% concordance among clinicians: treatment time > 12 months, 44%; treatment time between 6–12 months, 44%; treatment time < 6 months, 12%). High agreement (80.2%) among the clinicians was found regarding the biomechanics (surgery + orthodontics + skeletal anchorage, 79%; surgery + orthodontics, 19%; only orthodontics, 2%; only surgery, 0%). The most frequent reason for proposing M2M extraction was its depth and inclination (78.3%).

Primary outcome variable and clinical relevance.

Table 2 summarized descriptive statistics concerning the most appropriate radiological examination for M2M impaction. Except for M2M height to alveolar crest and M2M depth to the M1M, the other characteristics were better visualized using 3D examination with higher clinical relevance. Cone-beam CT influenced the clinical decision in all the considered items (Table 3).

**Table 2:** Descriptive statistics of the absolute and relative frequencies concerning the most appropriate radiological examination for M2M impaction

Clinical diagnosis or decision^*^	Clinician choices	Clinician responses (n)	Clinician Percentage (%)	3D report relevance
Which imaging modality would be most appropriate to diagnose impacted mandibular second molar?				
M2M Root relationship with IAN	2D 3D	3 147	2 98	NA 3.43
M2M height to alveolar crest	2D 3D	89 61	59.3 40.7	NA 2.69
M2M depth to the M1M	2D 3D	86 64	57.3 42.7	NA 2.95
M2M inclination to M1M	2D 3D	50 100	33.3 66.7	NA 3.22
M3M position to M2M	2D 3D	42 108	28 72	NA 3.13
M2M relationship to the lingual plate	2D 3D	2 148	1.3 98.7	NA 3.32

*M2M* mandibular second molar, *M1M* mandibular first molar, *M3M* mandibular third molar, *IAN* inferior alveolar nerve, *NA* not available

^*^The total of clinicians’ response was 150 (15 M2M × 10 clinicians)

**Table 3:** Descriptive statistics of the absolute frequencies concerning the clinical response after 2D and 3D radiological examination of M2M impaction

How did the CBCT influence the clinical decision?			
	Clinical choices	Clinical responses^*^	Entity of clinical relevance
Exposure and traction vs extraction	No change Change	9 141	NA 3.06
Placement of traction pad	No change Change	14 136	NA 3
Biomechanics	No change Change	13 137	NA 3.01
Patient and parents education	No change Change	16 134	NA 3.33
Additional treatment time estimate	No change Change	16 134	NA 2.59

*NA* not available

^*^The total of clinicians’ response was 150 (15 M2M × 10 clinicians)

### Secondary outcome variable

The ordinal numerical score was obtained by the sum of each single parameter: in this sample the median value was 12 (range: 7 – 18; 1st quartile: 11; 3rd quartile: 15). After computing the null distribution tertiles, the difficulty degree has been summarized as follows: 1) simple, when the sum of all parameters is less or equal than 11; 2) moderate, when the sum of all parameters ranges from 12 to 14; 3) complicated, when the sum of all parameters is higher or equal than 15.

This analysis confirmed that no evidence of a possible inter- or intra- group (surgeon/orthodontist) bias in score attribution occurred. Table 4 investigated on the possible predictors of the ordinal numerical scoring, according to the Poisson distributed generalised linear models: the model with the minimum AIC is the null model, defining that the score sum is neither predicted by the experts’ groups, nor by any specialist.

**Table 4:** Poisson distributed generalised linear model for investigating the possible inter-group (Group: surgeons vs orthodontists) or intra-group (Specialist: surgeons or orthodontists) bias in score attribution (covariate relation). The lowest AIC is highlighted in the null model (bold): the score sum is neither predicted by the experts’ groups, nor by any specialist

Covariate relation	AIC
Group OR Specialist	723.64
Group AND Specialist	721.97
Group	720.02
Specialist	720.02
**(Intercept)**	**718.04**

In Table 5 a multivariable analysis explores the possible predictive role of the anatomical and radiological features for the scoring system. Starting from an additive maximal model and proceeding by a top-down simplification procedure, a minimal adequate model has been disclosed. In the minimal adequate model, the significant contribution of alveolar crest (ACs), angle between M2M and M1M (M2M1o), mandibular third molar (M3Mo), and cortical plate (CPo) has been revealed. Specifically, evidence showed that the effect size of ACs (0.145) approximately doubles the other three effects (0.076, 0.098 and 0.083, respectively for M2M1o, M3Mo and CPo). Consequently, a new scoring classification system can be introduced, according to the simple algebraical relation:

**Table 5:** Multivariable analysis on the possible predictive role of the anatomical and radiological features for the scoring system

	Estimate	Std. Error	Z value	*p*-value
Intercept	1.78	0.12	14.46	< 0.0001*
Acs	0.15	0.05	3.22	0.001^*^
M2M1o	0.08	0.03	2.41	0.016*
M3Mo	0.09	0.04	2.74	0.006*
CPo	0.08	0.03	2.57	0.01*

^*^Statistically significant

*ACs* alveolar crest-surgical item, *M2M1o* angulation between M2M and M1M-orthodontic item, *M3Mo* mandibular third molar-orthodontic item, *CPo* cortical plate-orthodontic item

## *2 ACs* + *M2M1o* + *M3Mo* + *CPo*

**Fig. 3:**
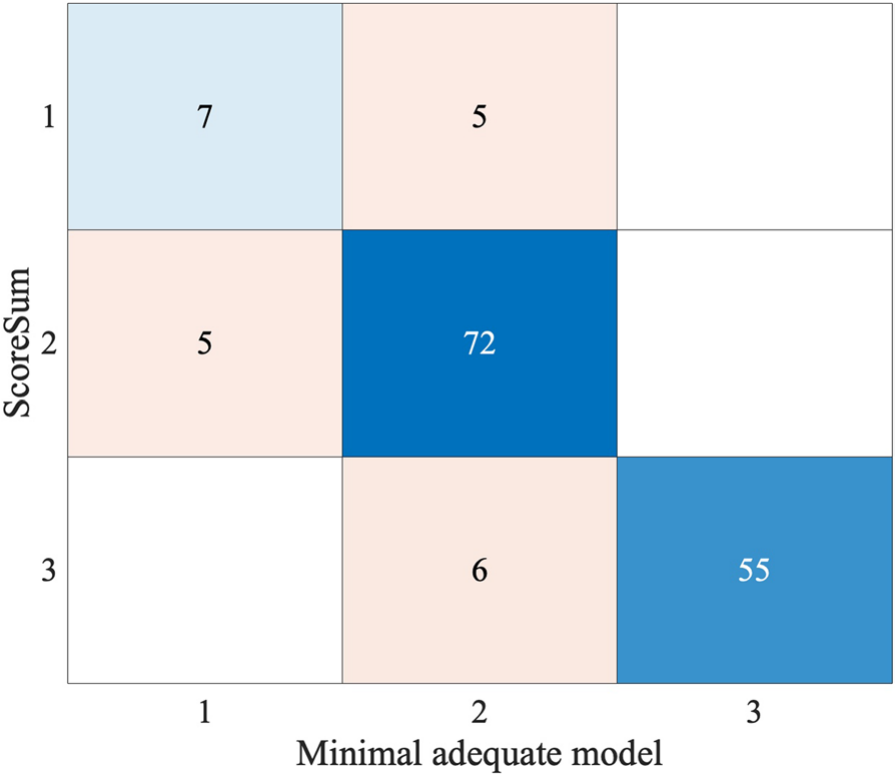
Confusion matrix showing the agreement between the score defined by the sum of each parameter of the classification and the minimal adequate model considering the significant contribution of alveolar crest (ACs), angle between M2M and M1M (M2M1o), mandibular third molar (M3Mo), and cortical plate (CPo)

And claiming the low-, middle-, and high- risk for M2M impaction when score < 8, score in [8, 11], score > 11, respectively. The confusion matrix highlights the agreement between the two proposed classification scores (Fig. 3).

## Discussion

This study aimed to introduce the first 3D classification of difficulty degree for M2M impaction, after describing the difference between 2 and 3D diagnostic records for the subsequent therapeutic choices. Precise diagnosis is imperative to ascertain the most suitable treatment among available therapeutic modalities and the preoperative assessment of M2M impaction require collaborative effort between oral surgeons and orthodontists to establish an accurate diagnosis and evaluate the complexity of each case [1, 19]. Identifying this condition promptly is crucial to initiate treatment at the opportune age [1, 11]. Early adolescence has been recognized as the optimal period for addressing impacted M2M due to incomplete root development and the third molar being in its germinal stage [10–12, 19, 20]. While some authors have reported favorable outcomes in adult patients, younger individuals have shown superior results with quicker improvement of their clinical condition [3, 7, 9–12, 16, 21]. As reported by La Monaca and colleagues, teeth movement in adolescents are easier and more appropriate than in adults who are often intolerant to the fixed orthodontic therapy [7]. The incomplete root apexification likely plays a significant role in the success of treatment, enabling the advantageous utilization of the residual eruptive force of the molar.

The initial findings of this analysis confirmed distinctions between 2 and 3D diagnostic records. Specifically, only the measurement of M2M height to the alveolar crest and M2M depth to the M1M exhibited satisfactory evaluation with orthopantomography. Conversely, for all other characteristics, the assessment favored the use of 3D examinations, offering superior visualization and greater clinical relevance. Undoubtedly, the three-dimensional analysis with the evaluation of M2M impaction in the three spatial axes is fundamental both for the surgeon, who must avoid intra-operative damage to the surrounding structures, and for the orthodontist, who has to manage the most favourable biomechanics. Cone-beam CT scans influenced clinical decisions across all examined aspects, particularly when considering the patients and their parents’ education. The final decision depends on diagnostic features, patient-related items, and operator-related skills. Diagnostic factors encompass the available diagnostic tools, the degree of impaction severity, the presence of any pathological bone abnormalities, and the condition of both affected and neighboring teeth. Patient-specific items include the patient's age and their cooperation during treatment. Operator-related factors involve the skills of the orthodontist in using the available tools, the surgeon's expertise in executing the most appropriate procedure, and their prior experience.

The therapeutic alternatives for treating impacted M2Ms could involve surgical techniques (surgical uprighting, surgical repositioning), orthodontic, or surgical-orthodontic approaches. It is crucial to preoperatively assess the risks and benefits of each treatment option [1, 6, 7, 12, 16, 19, 22–26]. Typically, impacted M2Ms are managed through a combination of surgical intervention and orthodontic traction, which has shown a high success rate, mainly in cases of moderate to severe impaction [1, 6]. In some cases, immediate success can be achieved solely through surgery, which repositions the M2M within the dental arch. However, orthodontic treatment is often needed to stabilize the repositioned tooth or refine the occlusal relationship. Conversely, orthodontic treatment only is frequently adopted when the inclination is limited and the M2M crown has already erupted. Otherwise, before orthodontic traction begins, surgical procedures can effectively remove mucosal and bone coverage, exposing the dental crown for proper appliance placement, can aid in dislocating the tooth, remove pathological lesions, and extract obstructing third molars if necessary [1]. M2M impaction can show a partial or total mucosal coverage, or a bone impaction for the most severe cases. A direct consequence of its depth is the relationship between M2M and the mandibular canal because a closer position with the tooth can result in alveolar nerve injuries during surgical manoeuvres. In particular, surgical uprighting, surgical repositioning, and extractions should be performed with caution because the tooth luxation could cause severe post-operative complications [1]. Orthodontically, the difficulty increases with a more horizontal position of M2M where a deeper surgical approach is mandatory, and a wider distal tipping of the tooth is necessary. As observed by most authors, the angulation can influence the therapeutic outcomes [7, 10]. Mesial inclination is the most common position for impacted M2M, mainly due to an abnormal eruption path [7, 10, 27]. This position often allows a less complex treatment for the M2M repositioning because the tooth usually has a potential of eruption yet [10]. As reported in literature, the angulation of impacted M2M usually ranged between 13° and 75° [28]. For this reason, 45° was considered the cut-off value for distinguishing M2M impaction from simple to moderate difficulty. Although some studies suggested that periodontal defects do not worsen after orthodontic uprighting of impacted M2Ms, a thorough preoperative evaluation is essential for guiding the decisions of both the surgeon and the orthodontist and may impact the outcome [1, 6, 29]. Their final evaluation should also include the buccal-lingual position in relation to the cortical plates. If M2M is buccally positioned or in the middle between buccal and lingual plates, a buccal access can be performed, with less surgical risk. On the contrary, in the complicated cases of transverse position, surgical treatment can require a lingual access or a double access (buccally and lingually), mainly in cases of tooth extraction. With these approaches, iatrogenic injury of the lingual nerve can compromise the therapeutic purpose. Orthodontically, anatomical and biomechanical considerations guide the difficulty assessment of impacted M2M. Because of cortical plates are the hardest part of the mandibular bone, higher forces are needed to achieve the tooth movement. Furthermore, it is mandatory to consider that, in the mandible, orthodontic traction can be applied only on the buccal side, providing better outcomes if M2M is parallel to the cortical walls and increasing the difficulty if it is oblique or completely perpendicular to them. Finally, a particular focus should be reserved to the posterior eruption space. An adequate retromolar space should be able to accommodate the lower second molar and the third molar is not always an obstacle for M2M recovery, mainly if it is completely distal to the impacted tooth [1, 7]. As reported by Kim et al., the development of the retromolar space can be predicted considering age and sex because it increases by 1.5 mm per year until 14 and 16 years of age for girls and boys, respectively [18]. Furthermore, as showed by Padwa et al., also surgical procedures could improve the mandibular bone remodelling in adolescents [6]. This anamnestic data can be very important during the diagnostic phase, influencing the clinical management.

**Fig. 4:**
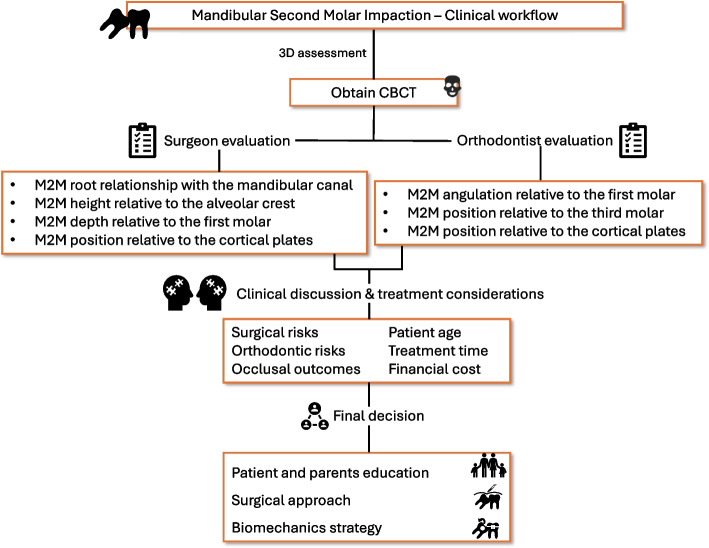
Clinical workflow of the decision-making process for impacted mandibular second molars

During the validation process of this new classification, the analysis confirmed the absence of any discernible inter- or intra-operator bias in score assignment. The assessment of M2M impaction was objectively conducted by both orthodontists and surgeons (Fig. 4). In the pursuit of a simplified method for calculating M2M difficulty, a minimal adequate model was proposed. This model introduces a new simplified scoring classification system, highlighting the significant impact of specific factors such as height compared to the alveolar crest, angle between M2M and M1M, relationship with the mandibular third molar, and relationship with the cortical plate. Despite potential statistical errors attributed to exponential calculations, this new scoring system enables clinicians to establish an easy algebraic relationship, categorizing M2M impaction into low-, middle-, and high-risk levels. This proposed approach is both straightforward and swift, demonstrating stronger agreement with the initial scoring method based on the summation of individual parameters. Clinically, an additional advantage of this model is its ability to provide consistent risk assessments, which aids in the planning of both surgical techniques and orthodontic biomechanics. This facilitates more informed decision-making and has the potential to improve patient outcomes by enabling more targeted treatment interventions.

The primary limitation of this study is the small sample size of this single-center study which serves as a pilot for validating the proposed classification system, stemming from the low incidence and prevalence of this clinical condition. Furthermore, statistical permutation tests were employed, confirming the statistical validity of these results due to the reduced variability of these data. Additionally, no data about treatment choices were reported, as this study solely focused on enhancing the diagnostic criteria for M2M impaction. This aspect is fundamental for obtaining detailed 3D information and improving consistency in diagnosis, communication, and outcome assessment among clinicians.

## Conclusion

In conclusion, three-dimensional evaluation of impacted M2Ms could enhance diagnostic accuracy, surpassing the limitations of 2D radiographic records. A 3D classification system was proposed and validated by a group of experienced surgeons and orthodontists, who play key roles in the treatment of this clinical condition. Future research should encompass prospective analyses involving larger, multicenter datasets to thoroughly assess the significance of this difficulty score system in guiding clinical management of M2M impaction. Furthermore, future avenues may explore the integration of machine learning and artificial intelligence to generate comprehensive data inputs, potentially aiding in diagnosis and, ideally, enhancing the final decision-making protocol.

## Supplementary Information

Supplementary Material 1.

Supplementary Material 2.

## Data Availability

The datasets used and/or analysed during the current study are available from the corresponding author [SB] on reasonable request.

## Abbreviations

3D: Three-dimensional
M2M: Mandibular second molar
2D: Bidimensional
AIC: Akaike information criterion
CBCT: Cone Beam Computed Tomography
M3M: Mandibular third molar
M1M: Mandibular first molar
MCs: Mandibular canal-surgical item
Acs: Alveolar crest-surgical item
M1Ms: Mandibular first molar-surgical item
CPs: Cortical plate-surgical item
M2M1o: Angulation between M2M and M1M-orthodontic item
M3Mo: Mandibular third molar-orthodontic item
CPo: Cortical plate-orthodontic item
IAN: Inferior alveolar nerve
