# Supplementary material for: Mandibular second molar impaction: introducing a novel and validated 3D classification system
Source: BMC Oral Health. 2024 Oct 10;24:1209. doi: 10.1186/s12903-024-05006-x (PMC11468259; doi:10.1186/s12903-024-05006-x)
Supplement: Supplementary file 1 — Supplementary Material 1. [file 12903_2024_5006_MOESM1_ESM.pdf]

Patient: Case n° _____, Age _________ years

Oral Surgeon ☐ Orthodontist ☐

1. Do the conventional diagnostic records provided indicate that lower third molar is a physical obstacle for the eruption of the lower second molar?

   ☐ yes ☐ no

2. Would you request a CBCT before finalizing your treatment plan?

   ☐ yes ☐ no

3. Will you attempt recovery for impacted mandibular second molar?

   ☐ yes ☐ no

4. If your answer to #3 is YES, please answer the following:

   a. How many months will you add to the treatment time estimate due to the mandibular second molar recovery for this patient?

   ☐ <6 months ☐ 6-12 months ☐ >12 months

   b. Please explain your approach (surgical and/or orthodontic) and the biomechanics for M2M recovery.

   ______________________________________________________________

   ______________________________________________________________

5. If your answer to #3 is NO, please explain your plan and the factors that led to this decision.

   ______________________________________________________________

   ______________________________________________________________
