# Supplementary material for: Mandibular second molar impaction: introducing a novel and validated 3D classification system
Source: BMC Oral Health. 2024 Oct 10;24:1209. doi: 10.1186/s12903-024-05006-x (PMC11468259; doi:10.1186/s12903-024-05006-x)
Supplement: Supplementary file 2 — Supplementary Material 2. [file 12903_2024_5006_MOESM2_ESM.pdf]

Expert Panel Survey

Item	What is the most appropriate radiological exam?		If CBCT, please specify the diagnostic relevance				
	OPG/TLL	CBCT+ diagnostic severity index	No importance 0	Low importance 1	Mild importance 2	Moderate Importance 3	High importance 4
Relationship between M2M roots and inferior alveolar nerve							
M2M position respect to alveolar crest							
M2M depth respect to M1M							
M2M inclination respect to M1M							
M3M position respect to M2M							
Relationship between M2M and the lingual plate							

How much did CBCT and the proposed severity index influence your clinical decision concerning the following items?	
	No 0 Low 1 Mild 2 Moderate 3 High 4
Exposure/ Traction/ Extraction	
Instructions for M2M traction position	
Biomechanics	
Patient and his parents' education	
Increase of treatment time	
